# Supplementary material for: Tuberculosis screening, isoniazid preventive therapy coverage and factors associated with active TB diagnosis among people living with HIV at public health facilities of central Ethiopia
Source: PLoS One. 2025 Mar 10;20(3):e0319676. doi: 10.1371/journal.pone.0319676 (PMC11892821; doi:10.1371/journal.pone.0319676)
Supplement: Attachment — Submitted filename: Author Response to Reviewewer comment TBHIV.docx [file pone.0319676.s002.docx]

**Author's response to Reviewers Comments**

Manuscript ID: - PONE-D-24-31425

Title of Manuscript: Tuberculosis Screening, Isoniazid Preventive Therapy, and Associated Factors Among People Living with HIV at Public Health Facilities of Central Ethiopia

Authors:

1. Mecha Aboma: abomamecha@gmail.com
2. Bayisa Abdisa
3. Gudata Imana
4. Kefalew Taye
5. Gonfa Moti
6. Merob Fufa

**A Point-by-point Response to Reviewer Comments to the Authors**

**Reviewer #1: Major Concerns:**

1. **Reviewer 1 Question 1**. Research Structure: The study appears to have two distinct components: the first focuses on patient progression through the early stages of the TPT cascade (screening for active TB, determining TPT eligibility, and providing TPT), while the second examines factors associated with active TB. The title does not fully reflect the second part of the study, and it seems somewhat disconnected from the first. It might be more coherent to analyze factors related to TB screening and TPT provision rather than factors associated with active TB.

**Authors Response 1.** Yes, the comment is accepted and revised and indicated by highlighted with track change as flow: Tuberculosis Screening and Isoniazid Preventive Therapy Coverage and Factors Associated with Active TB Diagnosis Among People Living with HIV at Public Health Facilities of Central Ethiopia

2. **Reviewer 1 Question 2.** Terminology: Some terms are used incorrectly, making the paper harder to understand. For instance, "TB infection" is used instead of "active TB," or "recurrent tuberculosis infection". Improving the language throughout the paper will enhance clarity.

**Authors Response 2.** Yes, the comment is accepted and corrected and indicated by highlighting with track change as flow: TB infection" is replaced by "active TB," or "recurrent tuberculosis infection"

3. **Reviewer 1 Question 3.** Conclusions Not Supported by Results: Several conclusions in the manuscript are not adequately supported by the data presented (lines 288-289, 296, 299-300). Please revisit these sections to ensure that the conclusions are based on the results.

**Authors Response 3**: Yes, the comment is accepted and revised and indicated by highlighted with track change lines 306-3010 and 3017-3025

**Minor Revisions:**

4. **Reviewer 1 Question 4.** Introduction: The introduction could be shortened, and it would be helpful to update the data, using statistics from 2021 and 2022 instead of 2018 and 2020.

**Authors Response 4**. Yes, the comment is accepted and revised and indicated by highlighted with track change, Lines 41-65, 69-70, 73-74, and 76-79

5. **Reviewer 1 Question 5.** Aim of the Study: The phrase "progress in reducing the risk of TB" (line 88) feels inappropriate as a research aim. A more accurate aim would be "progress in implementing TPT."

**Authors Response 5**. Yes, the comment is accepted and revised and indicated by highlighted with track change as flows: There is inadequate data on the extent to which progress has been made in implementing IPT among eligible people living with HIV, particularly in Ethiopia. Line 100-104

6. **Reviewer 1 Question 6**. Sample Size Calculation: The "design effect" concept used in sample size calculation (line 101) is unclear. Please provide further explanation or clarification.

**Authors Response 6**. Yes, the comment is accepted and revised and indicated by highlighted with track change as flows: To eliminate the design effect and have an adequate sample size, the sample size was doubled, and 10% of the total sample size was added to account for the non-response rate. The study was conducted in the West Shewa Zone, where 13 public health facilities were selected from 28 eligible health facilities located at the district level. People living with HIV (PLHIV) were then selected from the health facilities in each district.

7. **Reviewer 1 Question 7**. Figure 1: It would be more informative if the authors could display the overall number of PLHIV (People Living with HIV) in each facility and then provide the sample size for each. All the abbreviations should be added to the legend.

**Authors Response 7.** Yes, the comment is accepted, and the overall number of PLHIV (People Living with HIV) in each facility and the sample size for each health facility are included in Figure 1. And all the abbreviations added in the legend

8. **Reviewer 1 Question 8.** Patient Evaluation: It would be helpful to include information on how many patients had a positive symptom screening but did not complete further evaluation, as well as how many patients were found to have contraindications for TPT.

**Authors Response 8**. Yes, the comment is accepted and it was one of the limitations of this study, however contraindication for IPT was asked but all of the study participants responded that they had not reported any contraindication regarding IPT, however not included in the result.

9. **Reviewer 1 Question 9**. Diagnostic Methods: It is unclear why 551 patients were diagnosed with AFB smear while the initial screening was based on symptoms. Were all patients who were initially screened positive referred for AFB testing?

**Authors Response 9**. Thank you, the comment is accepted and corrected, indicated by highlighting with track change. It was a typographical error, corrected to 204 patients were diagnosed with AFB. Line 217, Table 2

10. **Reviewer 1 Question 10.** Typographical Error: There is a typographical error in the numbers; "796" is used instead of "769" in some calculations.

**Authors Response 10.** Thank you, the comment is accepted and corrected, indicated by highlighting with track change. It was a typographical error, corrected to 769. Table 2

11. **Reviewer 1 Question 11.** Table 3: I suggest restructuring Table 3. The second and third columns could be removed, and a column for p-values should be added to enhance the clarity of statistical significance.

**Authors Response 11.** The comment is accepted and corrected, indicated by highlighting with track change and P-Values are added

**Reviewer #2**

1. **Reviewer 2 Question 1.** The conclusion must mention the associated factors according to the title (‘Associated Factors Among People Living with HIV at Public Health Facilities of Central Ethiopia’).

**Authors Response 1.** The comment is accepted and corrected, indicated by highlighting with track change, and the associated factors were mentioned in the conclusion. Line 306-307

2. **Reviewer 2 Question 2:** This study still uses the AFB microscopy test to determine active TB. Is there no molecular rapid test available?

**Authors Response 2.** Yes, the comment is accepted. However, Acid-fast bacillus (AFB) tests and chest X-rays are common diagnostic tools used to detect TB infection among PLHIV at the studied health facilities (in the West Shew zone public health facilities) because molecular rapid tests were not available as indicated in the method line164-166.

3. **Reviewer 2 Question 3.** In data table 2, the CD4 variable (Current CD4 Count) should be added to group the CD4 count based on the diagnosis status of active or latent TB.

**Authors Response 3.** Yes, the comment is accepted. However, the Current CD4 count was already included in Table 2 and lines 218-120. Also, the National TB guideline in Ethiopia recommends IPT for all HIV-infected people who are unlikely to have active TB, regardless of CD4. CD4 count is not required to initiate Isoniazid Preventive Therapy (IPT) for people living with HIV (PLHIV) in Ethiopia. Similarly, Ethiopia adopted the “Universal Test and Treat” strategy to its national policy in 2016 to put all people living with HIV/AIDS (PLHIV) on antiretroviral therapy (ART) regardless of their World Health Organization (WHO) clinical stage or CD4 cell count level

4. **Reviewer 2 Question 4**. There were 257 (46.1%) eligible PLHIV patients who were not given IPT, please provide clear reasons for this.

**Authors Response 4**: Yes, the comment is accepted. However, most of these concerns were addressed in the discussion part lines 278-292, and in the result part mentioned as unknown reasons line 209. Because the study was focused only on the patients and did not address the factors related to the health facility as well as healthcare professionals, thus it could be one of the limitations of this study as further study including health facilities and healthcare professional-related factors was recommended in the conclusion part in line 321-325
