# Supplementary material for: Tuberculosis screening, isoniazid preventive therapy coverage and factors associated with active TB diagnosis among people living with HIV at public health facilities of central Ethiopia
Source: PLoS One. 2025 Mar 10;20(3):e0319676. doi: 10.1371/journal.pone.0319676 (PMC11892821; doi:10.1371/journal.pone.0319676)
Supplement: Attachment — Submitted filename: Author_Response_to_Reviewewer_comment_TBHIV_auresp_2.docx [file pone.0319676.s003.docx]

**2^nd^ Round Author's Response to Reviewers' Comments**

Manuscript ID: - PONE-D-24-31425

Title of Manuscript: Tuberculosis Screening, Isoniazid Preventive Therapy, and Associated Factors Among People Living with HIV at Public Health Facilities of Central Ethiopia

Authors:

1. Mecha Aboma: abomamecha@gmail.com
2. Bayisa Abdisa
3. Gudata Imana
4. Kefalew Taye
5. Gonfa Moti
6. Merob Fufa

**A Point-by-point Response to Reviewer Comments to the Authors**

**Reviewer #1: comments, Dear Authors,**

Thank you for addressing most of the comments and clarifying the questions raised in the previous review. The manuscript has improved significantly. However, there are still a few points that remain unaddressed, particularly regarding terminology and numerical inconsistencies (comment 2 and 10 from my previous review). I would like to bring up two additional comments for your consideration: Table 3 – It would be helpful to specify whether the reported p-values correspond to the crude or adjusted OR.

Lines 255-256 – Could you clarify whether you mean that the reported TB incidence was inflated due to false-positive AFB results? Typically, AFB testing is more prone to false negatives rather than false positives, which is the greater concern with this diagnostic method.

I appreciate your efforts in revising the manuscript and look forward to your response.

**Authors Response.** Thank you for thoroughly reviewing my paper and helping me improve its quality. Yes, all the comments are accepted and revised and indicated by highlighting with track changes as flows.

1. **Reviewer comment 1:** Comments 2 and 10 from my previous review).

**Authors Response 1**

- Yes, the comment is accepted and corrected and indicated by highlighting with track change as flow: TB infection" is replaced by "active TB throughout the document (Line 128).
- Thank you, the comment is accepted and corrected, indicated by highlighting with track change. It was a typographical error, corrected to 769 (Line 25, 420).

2. **Reviewer comment 2:** Table 3 – It would be helpful to specify whether the reported p-values correspond to the crude or adjusted OR.

**Authors Response 2**

- The comment is accepted and corrected, indicated in Table 3: The p-values correspond to the Adjusted OR and are specified in Table 3

3. **Reviewer comment 3**: Lines 255-256 – Could you clarify whether you mean that the reported TB incidence was inflated due to false-positive AFB results? Typically, AFB testing is more prone to false negatives rather than false positives, which is the greater concern with this diagnostic method.

**Authors Response 3**

Thank you, the comment is accepted and corrected, as indicated by highlighting with track change. In the first draft of the research paper, I submitted a sentence like this: “These differences might be attributed to **false negatives** reported for AFB testing and chest X-ray.” This typographical error likely occurred while I was editing and incorporating feedback from the editors and academic reviewers. I apologize for the typographical error; it has been corrected to "a false negative." Additionally, this statement pertains not only to the inflated active TB diagnoses reported in our study but also serves as a justification for the overall inconsistency of active TB diagnoses reported in our study compared to those reported by others, whether higher or lower than our findings. Thus, the comment has been genuinely accepted and corrected as flow (Line 255-259).

These differences might be attributed to false negatives reported for AFB testing and chest X-rays in diagnostic services. Additionally, inconsistent active TB diagnostics might result from inadequate diagnostic equipment, poor microscopy quality, untrained healthcare providers, substandard laboratory practices, and insufficient quality control measures, particularly in resource-limited settings.
